# The AF-1-deficient estrogen receptor ERα46 isoform is frequently expressed in human breast tumors

**DOI:** 10.1186/s13058-016-0780-7

**Published:** 2016-12-07

**Authors:** Elodie Chantalat, Frédéric Boudou, Henrik Laurell, Gaëlle Palierne, René Houtman, Diana Melchers, Philippe Rochaix, Thomas Filleron, Alexandre Stella, Odile Burlet-Schiltz, Anne Brouchet, Gilles Flouriot, Raphaël Métivier, Jean-François Arnal, Coralie Fontaine, Françoise Lenfant

**Affiliations:** 1 INSERM U1048, Institut des Maladies Métaboliques et Cardiovasculaires, Université de Toulouse, BP 84225, 31 432 Toulouse cedex 04, France; 2 Pôle IUC Oncopole CHU, Institut Universitaire du Cancer de Toulouse - Oncopole, 1 avenue Irène Joliot-Curie, 31059 Toulouse cedex 9, France; 3 UMR CNRS 6290, Institut de Genétique et Développement de Rennes, Equipe SP@RTE, Rennes, 35042 Cedex France; 4 PamGene International B.V, P.O. Box 1345, 5200 BJ ‘s-Hertogenbosch, The Netherlands; 5 Institut de Pharmacologie et de Biologie Structurale, Université de Toulouse, CNRS, UPS, Toulouse, France; 6 INSERM U1085, IRSET (Institut de Recherche en Santé, Environnement et Travail), Université de Rennes 1, 35000 Rennes, France

**Keywords:** Breast cancer, Estrogen receptor ERα, Isoforms, Diagnosis, Internal ribosomal entry site, Activation function

## Abstract

**Background:**

To date, all studies conducted on breast cancer diagnosis have focused on the expression of the full-length 66-kDa estrogen receptor alpha (ERα66). However, much less attention has been paid to a shorter 46-kDa isoform (ERα46), devoid of the N-terminal region containing the transactivation function AF-1. Here, we investigated the expression levels of ERα46 in breast tumors in relation to tumor grade and size, and examined the mechanism of its generation and its specificities of coregulatory binding and its functional activities.

**Methods:**

Using approaches combining immunohistochemistry, Western blotting, and proteomics, antibodies allowing ERα46 detection were identified and the expression levels of ERα46 were quantified in 116 ERα-positive human breast tumors. ERα46 expression upon cellular stress was studied, and coregulator bindings, transcriptional, and proliferative response were determined to both ERα isoforms.

**Results:**

ERα46 was expressed in over 70% of breast tumors at variable levels which sometimes were more abundant than ERα66, especially in differentiated, lower-grade, and smaller-sized tumors. We also found that ERα46 can be generated via internal ribosome entry site-mediated translation in the context of endoplasmic reticulum stress. The binding affinities of both unliganded and fully-activated receptors towards co-regulator peptides revealed that the respective potencies of ERα46 and ERα66 differ significantly, contributing to the differential transcriptional activity of target genes to 17β estradiol (E2). Finally, increasing amounts of ERα46 decrease the proliferation rate of MCF7 tumor cells in response to E2.

**Conclusions:**

We found that, besides the full-length ERα66, the overlooked ERα46 isoform is also expressed in a majority of breast tumors. This finding highlights the importance of the choice of antibodies used for the diagnosis of breast cancer, which are able or not to detect the ERα46 isoform. In addition, since the function of both ERα isoforms differs, this work underlines the need to develop new technologies in order to discriminate ERα66 and ERα46 expression in breast cancer diagnosis which could have potential clinical relevance.

**Electronic supplementary material:**

The online version of this article (doi:10.1186/s13058-016-0780-7) contains supplementary material, which is available to authorized users.

## Background

Breast cancer is a major public health concern because its incidence continues to rise. It is the second most common cancer overall and by far the most frequent cancer among women [1]. The etiology of breast cancer is multifactorial, and although the mechanisms of carcinogenesis remain poorly defined the role of hormones is recognized as a major risk factor in breast cancer development, in particular 17β estradiol (E2) and its derivatives.

Estrogen receptor (ER)α is one of two ERs and is involved in several key aspects of breast cancer diagnosis [2]. Firstly, ERα protein immunoreactivity in the nucleus of mammary epithelial cells is systematically evaluated and quantified during anatomopathological diagnosis, with 70% of breast cancers initially described as ERα-positive [2]. Secondly, ERα expression in breast cancers correlates with improved survival rates and reduced risk of recurrence and metastases [3–5]. Finally, the blockade of ERα activity represents a major targeted therapy for ERα-positive breast cancer, with tamoxifen and aromatase inhibitors having already benefitted millions of women [6]. Despite the success of these treatments, 30 to 40% of patients develop resistance [7]. This highlights the need for further in-depth characterization of ERα-positive tumors and a full understanding of the mechanisms underlying the disease in order to propose new therapeutic approaches.

**Fig. 1:**
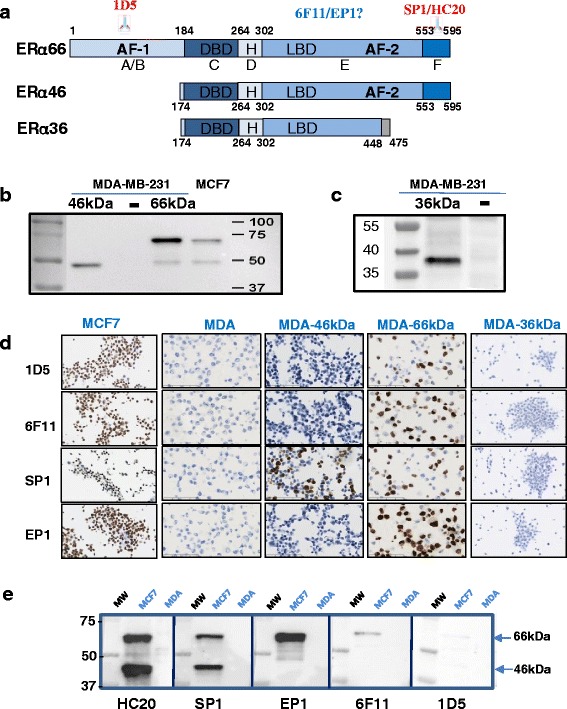
Recognition of estrogen receptor alpha (*ERα*) isoforms by antibodies used for human breast cancer diagnosis. **a** Schematic representation of the ERα66, ERα46, and ERα36 isoforms. The location of the known epitopes used for the generation of antibodies is indicated. **b** Representative Western blot analyses with the SP1 antibody on extracts from MDA-MB-231 cells transfected with plasmids encoding either ERα46 (MDA-46 kDa) or ERα66 (MDA-66 kDa) and from MCF7 cells which express both isoforms, or **c** with the Anti-Flag antibody on extracts from MDA-MB-231 cells transfected with plasmids encoding the ERα36 isoform. **d** The different antibodies used in breast cancer diagnosis (1D5, 6 F11, SP1, and EP1) were tested for their ability to recognize either ERα66, ERα46, or both isoforms in immunocytochemistry experiments performed in MCF7, MDA-ERα46, and MDA-ERα66 or MDA-ERα36 cells. **e** Representative picture of Western blot experiments evaluating the expression of both ERα isoforms in MCF-7 cells, as determined by the different antibodies indicated. Protein extracts prepared from MDA cells were used as an ERα-negative control. *AF* activation function, *DBD* DNA-binding domain, *LBD* ligand-binding domain

In addition to the “classic” full-length 66-kDa ERα (ERα66) which harbors the two activation functions, AF-1 and AF-2, two other isoforms of 46 kDa (ERα46) and 36 kDa (ERα36) have been characterized. ERα36 differs from ERα66 by lacking both transcriptional activation domains (AF-1 and AF-2) and encoding a unique 29 amino acid sequence [8]. In contrast, ERα46 only lacks the first 173 N-terminal amino acids which harbors AF-1 and is thus completely identical to the amino acids 174 to 595 of ERα66 (Fig. 1a). ERα46 has been reported to be expressed in various cell types such as human osteoblasts [9], macrophages [10], and vascular endothelial cells [11], but also in cancer cells such as colorectal tumor tissues [12] and tamoxifen-resistant breast cancer cell lines [13]. Mechanisms regulating both the expression of ERα46 and its functions remain essentially unknown. It can be generated by either alternative splicing [14], proteolysis [15], or an alternative initiation of translation via an internal ribosome entry site (IRES) [16]. This latter mechanism generates two different proteins from a single RNA. A few studies have suggested that ERα46 plays an inhibitory role in the growth of cancer cell lines, suggesting that ERα46 could affect tumor progression. The overexpression of ERα46 in proliferating MCF7 cells provoked cell cycle arrest in G0/G1 phase and inhibited ERα66-mediated estrogenic induction of the AF-1-sensitive reporters c-fos and cyclin D1, as well as estrogen-responsive element (ERE)-driven reporters [14, 17]. It was also shown that ERα46 inhibits growth and induces apoptosis in human HT-29 colon adenocarcinoma cells [12]. This inhibition likely occurs through competition between ERα66 and ERα46 homodimers and heterodimers for binding to the ERE [17]. The role of the AF-1-deficient ERα46 isoform has also been questioned in vivo using mice deficient in the ERα A/B domain (named *ER*α*AF-1* ^*0*^), which express only a short 49-kDa isoform that is functionally similar to ERα46. These *ER*α*AF-1* ^*0*^ mice revealed a complete infertility phenotype [18] that was associated with an altered proliferative effect of E2 on the uterine epithelium and a loss of its transcriptional response in this tissue [19].

Thus, the roles and functions of this ERα46 isoform appear to be different from those of full-length ERα66. The expression level of this truncated isoform in human breast tumors remains unknown, even though the expression of a 47-kDa isoform of ERα in human breast cancers was reported more than two decades ago [20]. Currently, several antibodies are used for immunohistochemical detection of ERα in human breast tumor diagnosis but most of them have not yet been thoroughly characterized in terms of ERα46 recognition.

In this study, we first characterized the various antibodies commonly employed in immunohistochemical diagnosis for their ability to detect ERα46. We then analyzed the relative expression of the ERα isoforms in a panel of 116 ERα-positive breast tumors. We also examined the mechanism of ERα46 generation and its specificities in term of coregulator binding and of functional activities.

## Methods

### Cell culture

MDA-MB231 and MDA-MB231 cells stably expressing ERα66 (MDA-ERα66; [21]) or ERα46 (MDA-ERα46; [22]) or transiently transfected with pSG5Flag-ERα36 (kindly provided by M. Le Romancer) were maintained in Dulbecco’s modified Eagle’s medium (DMEM; Sigma) supplemented with 5% fetal calf serum (FCS; Biowest) and antibiotics (Sigma) at 37 °C under 5% CO_2_. Media used to maintain cells expressing either ER isoforms also included hygromycin (Calbiochem; 0.8 mg/ml). We also transiently transfected MDA cells with either the pcDNA3.1 plasmid, encoding the cDNA of ERα46, or a mutated version, ERα46kDa^0^, using the *TransIT*®-BrCa Transfection Reagent (Euromedex, France). This mutated plasmid was obtained by subcloning a fragment from ERα66 that contains an AUG/UCG mutation at codon 174 and an AUG/AUA mutation at codon 176 [16]. Stably transfected MCF7 sub-clones MCF7-B0 (control) and MCF7-B1 and MCF7-B2 overexpressing ERα46 were obtained by transfecting MCF7 cells with pcDNA6/TR plasmid and pcDNA4/TO expression vector containing or not the ERα46 cDNA sequence (T-Rex system, Invitrogen) with jetPEI reagent (Polyplus transfection). The clones were selected with 5 μg/ml blasticidin and 100 μg/ml zeocin (Invitrogen). Individual clones were isolated and grown in a medium containing selective antibiotics to maintain selection pressure. Expression of the proteins of interest was induced by a 48-h treatment of MCF7 sub-clones with 1 μg/ml tetracyclin.

### Immunohistochemistry

Cells were formalin-fixed and paraffin-embedded using the Shandon™ Cytoblock™ Cell Block Preparation System, according to the manufacturer’s protocol. Immunohistochemistry was performed with a Dako Autostainer Link 48 on 3-μm sections. Antigen retrieval was performed using a Dako PT Link pressure cooker in pH 6.0 citrate buffer. An EnVision™ system was used for antibody detection. The anti-Flag (M2; Sigma-Aldrich) and a panel of anti-ERα antibodies (SP1 (Abcam), HC20 (SantaCruz), 6 F11 (Novocastra), 1D5 and EP1 (Dako)) were used.

### Human breast cancer sample collection

The retrospective study used tumors samples from patients diagnosed with invasive breast carcinoma, established as being ERα-positive on a previously performed biopsy (see Additional file 1 (Figure S1) for clinical parameters of the patients used). The diagnosis was performed with the 6 F11 or 1D5 antibodies between 2011 and 2014. Tumors were frozen in 1.5-ml cryotubes using the SnapFrostII™ fast freezing system (Excilone, France) and stored at –80 °C. Patients with an ipsilateral recurrence of breast cancer who had received neoadjuvant chemotherapy or chemotherapy treatment for another disease or who received thoracic radiation therapy (recurrence or another pathology) were excluded. All tumors were classified by the anatomopathologist (human epidermal growth factor receptor-2 (HER2) status, tumor size, ERα overexpression, lymph node involvement, histological type) and were graded according to Elstone and Ellis’ guidelines [23]. The analysis was performed on a series of 116 ERα-positive invasive ductal or lobular breast carcinomas (22 grade I, 60 grade II, and 34 grade III).

### Western blots on tumor samples

Samples of each tumor were lysed in cold lysis buffer (150 mM NaCl, 50 mM Tris-HCl pH 7.5, 0.1% SDS, 1 mM ethylenediaminetetraacetic acid (EDTA), 5 mM NaF, 1 mM orthovanadate, 0.5 mM 1,4-dithiothreitol (DTT), and protease inhibitors) using a Precellys Homogeniser (Bertin Technologies, France). Proteins were separated on a 10% SDS-PAGE gel and transferred to a nitrocellulose membrane. After blocking, membranes were incubated overnight with the primary antibody (anti-ERα (SP1) or anti-GAPDH (6C5); Santa Cruz). Subsequently, blots were incubated with a horseradish peroxidase (HRP)-conjugated secondary antibody (anti-rabbit; Cell Signaling) and visualized by enhanced chemiluminescence (ECL) detection according to the manufacturer's instructions (Amersham Biosciences, CT) using a ChemiDoc™ Imaging System (Bio-Rad). Bands corresponding to ERα46 and ERα66 were quantified using ImageJ densitometry, and the ratio of the band intensities was calculated.

### Endoplasmic reticulum stress

MDA-66 kDa or MCF7 cells were seeded in six-well plates. At 80% confluence, the cells were subjected to 6 h of stress with DTT (Euromedex) or thapsigargin (Sigma) as indicated, then rinsed in phosphate-buffered saline (PBS), lysed in SDS buffer (5% SDS, 10% glycerol, 80 mM Tris pH6.8), and sonicated. Western blot analyses were then performed on 4–20% denaturing polyacrylamide “stain-free” gels (BioRad).

### Immunoprecipitation and proteomic analysis

ERα-enriched protein fractions from tumor protein extracts were obtained through immunoprecipitation using the anti-human ERα primary HC20 antibody. Following their purification using Protein G sepharose beads, a first Western blot was performed to check the efficiency of the immunoprecipitation. In parallel, the immunoprecipitate was diluted with Laemmli buffer, then separated by SDS-PAGE using a short and low-voltage electrophoretic migration. After Instant Blue staining, the bands corresponding to ERα46 and ERα66 were respectively excised from the gel. Proteins were in-gel digested by trypsin, and resulting peptides were extracted from the gel and analyzed by nano-liquid chromatography coupled to tandem mass spectrometry (LC-MS/MS) using an ultimate 3000 system (Dionex, Amsterdam, Netherlands) coupled to an LTQ-Orbitrap Velos mass spectrometer (Thermo Scientific, Bremen, Germany).

The LTQ-Orbitrap Velos was operated in data-dependent acquisition mode with the Xcalibur software. Survey scan MS spectra were acquired in the Orbitrap in the 300–2000 *m*/*z* range with the resolution set to a value of 60,000. The twenty most intense ions per survey scan were selected for collision-induced dissociation fragmentation, and the resulting fragments were analyzed in the linear ion trap (LTQ, parallel mode, target value 1e4). Database searches from the MS/MS data were performed using the Mascot Daemon software (version 2.3.2, Matrix Science, London, UK). The following parameters were set for creation of the peak lists: parent ions in the mass range 400–4500, no grouping of MS/MS scans, and threshold at 1000. Data were searched against SwissProt 20130407. Mascot results were parsed with the in-house developed software MFPaQ version 4.0 (Mascot File Parsing and Quantification) (http://mfpaq.sourceforge.net/) and protein hits were automatically validated with a false discovery rate (FDR) of 1% on proteins and 5% on peptides (minimum peptide length of six amino acids).

### Plasmids, lentiviral production, and luciferase assay

cDNA coding for the A/B (amino acids 2–173) domain of the human *ESR1* gene encoding ERα was amplified by polymerase chain reaction (PCR) and cloned into the *Spe*I and *Nco*I sites of the pTRIP CRF1AL2 bi-cistronic vector that encodes both the Renilla luciferase (LucR) and Firefly luciferase (LucF2CP) genes separated by this putative IRES-ERα sequence [24]. The final construct was verified by sequencing. In such a transgene, LucR expression is cap-dependent whereas LucF expression is IRES-dependent; thus, the level of IRES activity can be deduced from the LucF/LucR ratio. The production of lentiviral particles was performed in HEK293 cells. Transduced MDA-MB 231 cells (MDA-A/B) were subjected to ER stress as indicated. To test whether the stress-induced increase in LucF activity was not due to the generation of mono-cistronic LucF transcripts via an internal promoter or cryptic splicing, MDA-Lenti-AB (1/10) cells were exposed to two siRNAs-lucR and treated with 5 mM DTT or 100 nM thapsigargin. As control, cells were treated with scrambled siRNA. After a PBS wash, cells were frozen at –80 °C. Luciferase measurements were performed with a LB960 luminometer (Berthold) using the dual reporter assay kit (E1960; Promega) according to the manufacturer’s recommendations.

### RT-qPCR

MDA-MB231, MDA-ERα46, and MDA-ERα66 cells were plated in 9-cm diameter dishes in DMEM/0.5% charcoal-stripped FCS (csFCS) containing appropriate antibiotics in order to reach confluency 3 days later. Cells were then treated with 10^–8^ M E2 final for 4 h or with a similar volume of ethanol (vehicle). Total RNAs were then purified using the Trizol™ reagent (Life Technologies, Inc.) according to the manufacturer’s instructions. RNA (2 μg) was used as a template for reverse transcription (RT) by the M-MLV reverse transcriptase (Invitrogen) and Pd(N)6 random hexamers (Amersham Pharmacia Biosciences). Quantitative PCR used on 2 μl of 1/10th diluted RT reactions and 1 μM of specific oligonucleotides and were performed on BioRad CFX96 machines using BioRadiQ SYBR Green supermix with 50 rounds of amplification followed by determination of melting curves. Primers for RT-PCR were designed under the QuantPrime design tool (http://www.quantprime.de [25]). Independent triplicate experiments were conducted twice, and all values were normalized to *Rplp0* mRNA. Significant variations were evaluated using the GraphPadPrism™ software.

### Coregulator-peptide interaction profiling

Ligand-mediated modulation of the interactions between the ERα46 and ERα66 proteins and their coregulators was characterized by a MARCoNI (Microarray Assay for Real-time Coregulator-Nuclear receptor Interaction; PamGene International BV, the Netherlands). This method has been described previously [26, 27]. Briefly, each array was incubated with a reaction mixture of crude lysates from MDA-MB-231 cells stably expressing each isoform of ERα46 or ERα66 on buffer F (PV4547; all Invitrogen) and vehicle (2% DMSO in water) with or without the receptor ligands at the indicated concentrations. ERα66 was quantified by enzyme-linked immunosorbent assay (ELISA; Active Motif, USA) and ERα46 was normalized to ERα66 by Western blot analyses. SP1 antibody which specifically recognized both isoforms was used to detect the ERα bound on the PamChip microarray. For both ERα46 and ERα66 receptors, a dose-response curve was performed from 10^–12^ to 10^–7^ M E2 to directly compare their response to E2. For measurements of antagonist effects with 4-hydroxytamoxifen and Fulvestrant, 6.3 nM ( 10^–8.2^M) E2 was applied since both receptors were fully active at that concentration. Incubation was performed at 20 °C in a PamStation96 (PamGene International). Receptor binding to each peptide on the array was detected by SP1 antibody. The secondary anti-rabbit antibody conjugated to fluorescein and the goat anti-mouse antibody conjugated to fluorescein were used and given a fluorescent signal, which was further quantified by analysis of .tiff images using BioNavigator software (PamGene International).

### Statistical analyses

Comparisons between groups were performed using the Mann-Whitney rank sum test for continuous variables. Correlations between continuous variables were evaluated using the Spearman's rank correlation test. All *P* values are two-sided. For all statistical tests, differences were considered significant at the 5% level. Statistical analyses were performed using the STATA 13.0 software (STATA Corp, College Station, TX) or GraphPad Prism v.5.

## Results

### Characterization of the anti-ERα antibodies commonly used for breast tumor diagnosis

Apart from lacking the A/B domain and thus the AF-1 transactivation function, the ERα46 isoform is completely identical to the ERα66 isoform (Fig. 1a). Therefore, to characterize the expression of ERα46 in breast tumors, an antibody must be used that is directed against the C-terminal domain. This excludes 1D5, one of the first monoclonal antibodies to be available against ERα for tumor diagnosis [28], targeting an epitope in the A/B domain (Fig. 1a). Later on, the respective murine and rabbit monoclonal antibodies 6 F11 and SP1, with improved specificities compared to the 1D5 clone, became extensively used for diagnosis [29, 30]. More recently, the monoclonal rabbit antibody EP1 was commercialized. However, whereas the SP1 epitope is known to be in the C-terminal domain, the abilities of the 6 F11 and EP1 antibodies to recognize ERα46 have not, to our knowledge, been reported.

To test this, we used control ERα-negative MDA-MB-231 cells and MDA cells engineered to stably express either the ERα46 or ERα66 isoform or to transiently express the Flag-tagged ERα36 protein, alongside MCF7 cells co-expressing both proteins (Fig. 1b and c, and Additional file 1: Figure S2).

Interestingly, a small amount of ERα46 expression was found in MDA 66-kDa cells, presumably due to an alternative initiation of translation at Met 174 and/or Met 176 as previously suggested [16]. Immunocytochemistry performed on these five cell lines demonstrated that, among the four tested antibodies (1D5, 6 F11, SP1, and EP1), only SP1 was able to specifically detect the ERα46 isoform in the MDA 46-kDa cells (Fig. 1e). Of note, none of these antibodies was able to recognize the ERα36 isoform by immunocytochemistry.

The immunoreactivity of the different antibodies was also tested by immunoblotting with the HC-20 antibody, which is frequently used in Western blot analyses, but not for diagnosis since it is a polyclonal rabbit antibody (Fig. 1e). Whereas EP1 and 6 F11 only detected ERα66, the HC-20 and SP1 antibodies recognized both ERα46 and ERα66, which is well in line with the immunocytochemistry results. We also noticed that the 1D5 antibody had a quasi-undetectable reactivity when used in this procedure. Altogether, these data demonstrate that from the set of antibodies commonly used for breast cancer diagnosis, the SP1 antibody is the only one able to recognize the ERα46a isoform by immunohistochemistry.

### Quantification of ERα46 in human breast carcinomas

**Fig. 2:**
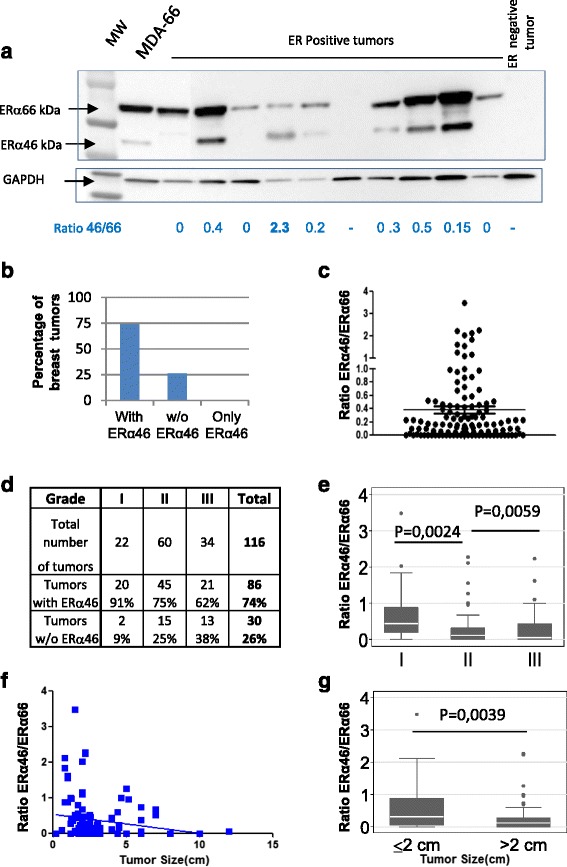
Evaluation of the relative expression of estrogen receptor alpha (*ERα*)46 and ERα66 in human ERα-positive breast tumors. **a** Representative image of Western blot of human ERα-positive breast tumor samples blotted with an anti-ERα antibody (SP1). One result representative of an ERα-negative tumor is also shown. The MDA-ER66 cell line that co-expresses ERα66 and ERα46 was used as a positive control. The ERα46/ERα66 expression ratio is indicated below each lane. **b** The summarized data expressed as percentages of tumors expressing ERα46 or not. **c** Distribution of the ERα46/ERα66 expression ratio among the 116 tumor samples. **d** The number and grade of tumors expressing ERα46. **e** The ERα46/ERα66 expression ratio depending upon tumor grade (I, II, or III). **f**, **g** The ERα46/ERα66 expression ratio in correlation to tumor size. The whiskers in the boxplots indicate the 10–25% and 75–90% intervals

Using SP1 antibody, we next performed Western blotting of 116 ERα-positive breast tumor samples (initially characterized with the 6 F11 or 1D5 antibodies) to compare the relative abundance of ERα46 and ERα66. Patients included in this study had not have received any neoadjuvant therapy. Most of the breast tumors (70%) expressed both isoforms, though at varying levels (Fig. 2a and b). The ERα46/ERα66 ratio varied from 0 to 3.48, with a mean average of 0.37 and a median of 0.16. Furthermore, even though the vast majority of tumors expressed lower levels of ERα46 than ERα66, 10% of the tumors tested expressed predominantly the shorter isoform (Fig. 2c).

We next analyzed the relationship between clinical parameters (grade and size of tumor) and ERα46 expression. We found that high-grade tumors correlated with lower ERα46 expression since 91% of grade I tumors expressed ERα46, whereas this figure was 75 and 62% for grades II and III, respectively (Fig. 2d). Moreover, the ERα46/ERα66 ratio of the relative expression of these isoforms was also significantly higher in low-grade tumors compared to tumors of grades II and III which are highly dedifferentiated (*P* = 0.0024 and *P* = 0.0059, respectively; see Fig. 2e). The abundance of ERα46 was also inversely correlated with tumor size (Fig. 2f). Finally, we classified our samples using a size parameter usually used by the American Joint Committee on Cancer (AJCC) to characterize tumor evolution, which is set at a 2-cm cut-off. Using this classification, we found that ERα46 expression was higher in small-sized tumors compared to tumors greater than 2 cm in diameter (*P* = 0.0039; Fig. 2g). Interestingly, even though there was a few HER2-positive tumors among our samples, a significant correlation was found between HER2 expression and expression of ERα46, indicating that HER2-positive tumors have low abundance of ERα46 (Additional file 1: Figure S3). All other parameters, including necrosis, were not significant.

A few studies have shown that 8% of tumors diagnosed as ERα-negative using the 1D5 antibody were actually positive for ERα when tested with next-generation antibodies such as SP1 [29–31]. The authors did not take into account the presence of ERα46, which cannot be detected by the 1D5 antibody. We therefore explored the possibility that these tumors may not express ERα66 but only ERα46 by evaluating the expression of the ERα46 isoform in a series of 19 tumors identified as ERα-negative using the 6 F11 antibody. However, none of these samples were found to express the short ERα46 isoform. A representative sample is shown in Fig. 2a. This study remains preliminary and should be extended to a larger sample series (in process).

Altogether, these data obtained by analyzing the expression of ERα46 in a panel of 116 ERα-positive breast tumors highlights the fact that ERα46 was expressed in more than 70% of cases. Furthermore, although the expression of this short isoform was highly variable, it correlated with the tumor evolution stage with a higher expression in low-grade tumors and lower expression in tumors that were larger, less differentiated, and of higher-grade.

### Identification of the ERα46 isoform

Although the bands observed by Western blot analysis were of the expected sizes, we wanted to confirm the nature of the detected proteins. To reach this aim, we purified the ERα proteins from MCF7 cells and from lysates of four tumor samples by immunoprecipitating the two ERα isoforms using the anti-human ERα primary HC20 antibody (Additional file 1: Figure S4A). After separation by SDS-PAGE, the gel bands corresponding to the 46-kDa and 66-kDa proteins were excised and further digested for proteomic analysis. In MCF7 cells (Table 1), 24.4% of the ERα66 sequence was covered, including a peptide in the N-terminal domain of amino acids 9–32. Importantly, and as expected, although 23.3% of the ERα46 sequence was detected, no peptide from the N-terminal A/B domain was identified. Proteomic analysis of immunoprecipitated ERα proteins from four tumor samples respectively covered 25% of the ERα66 sequence and 15.3% of the ERα46 sequence (Additional file 1: Figure S4B and S4C). Again, although peptides 184–206, 402–412, and 450–457 were found in the 46 kDa-sized band, no peptides located before the ATG at codon 174 were detected. The first peptide found is 184-206. Therefore, although we were unable to characterize the start codon of ERα46, we confirm for the first time that the 46-kDa band identified in Western blot analyses of ERα-positive tumors is without doubt a shorter isoform of ERα.

**Table 1:** Proteomic analysis of the 46-kDa protein detected in tumor samples

Tumor	Sample	Mascot score	Cov. (%)/isoform	No. of identified peptides	First N-terminal peptide identified and validated	Peptide identified before Met 174	Last C-terminal peptide identified and validated
MCF7	IP46	759	23.3	14	212–231	No	556–581
	IP66	440	24.4	12	009–032	Yes	556–581
Tumor 1	IP46	96	8.0	5	402–412	No	556–581
	IP66	52	4.4	1	556–581	No	556–581
Tumor 2	IP 46	228	15.3	5	184–206	No	556–581
	IP 66	522	25.0	10	38–48	Yes	556–581
Tumor 3	IP 46	65	3.0	1	450–467	No	450–467
Tumor 4	IP 46	35	1.8	1	402–412	No	402–412

Proteomic analysis results from the different cell lines and the four breast tumor samples

The % of sequence coverage corresponds to the number of amino acid residues identified in the proteomic analysis divided by the total number of amino acid residues in the protein sequence. The Mascot score is described in [52]. It uses statistical methods to assess the validity of a match. This enables a simple rule to be used to judge whether a result is significant or not. We report scores as -10*LOG10(P), where P is the absolute probability. A probability of 10^-20^ thus becomes a score of 200

### ERα46 can be expressed following alternative initiation of translation in response to stress

**Fig. 3:**
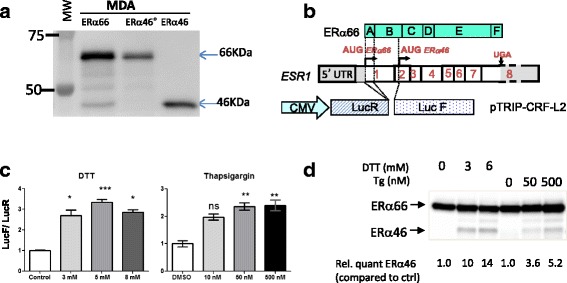
IRES-mediated generation of estrogen receptor alpha (*ERα*)46. **a** A representative image of a Western blot of extracts from MDA-MB-231 cells transfected with constructs expressing either ERα46, wild-type ERα66, or an ERα66 cDNA harboring mutated ATG174/176 codons (*ERα46* ^*0*^), illustrating the loss of expression of ERα46 in cells transfected with the vector encoding ERα46^0^. **b** Scheme illustrating the bi-cistronic pTRIP-hERaAB-L2 vector harboring the Renilla luciferase (*LucR*) and Firefly luciferase (*LucF(2CP)*) cistrons separated by the sequence coding for the A/B domains of the human ERα66. **c**, **d** Stress-induced alternative initiation of translation. **c** MDA-MB-231 cells transduced with lentivirus expressing the LucR-hERaAB-LucF construct were subjected to 6 h of stress with 1,4-dithiothreitol (*DTT*; 3, 5, or 8 mM), thapsigargin (*Tg*; 10, 50, or 500nM) or vehicle control (*ctrl*). Reporter gene activities from three independent experiments in duplicates are shown as the LucF/LucR ratio. **d** Representative Western blot of the increased expression of ERα46 observed in stressed MDA-MB-231 cells transfected with cDNA expressing ERα66. *P < 0.05, **P < 0.01, ***P < 0.001, versus control (Kruskal-Wallis) *ns* not significant

It has already been proposed that an alternative initiation of translation could participate in ERα46 generation through an IRES and the presence of two other potential initiation codons (AUG174/176) in the mRNA coding sequence for amino acids 2–173 of ERα [16]. In line with this potential mechanism, we were able to detect ERα46 by Western blot analysis in MDA cells transfected with full-length ERα66 (Fig. 1b). In order to definitively confirm this hypothesis, we analyzed ERα46 expression in MDA cells transfected with an ERα46 construct in which the two potential initiation codons of ERα46 (AUG174/176) were mutated (ERα46^0^). As shown in Fig. 3a, ERα46 expression was not detected using this ERα46^0^ construct, demonstrating that the two potential initiation codons are necessary to generate the ERα46 isoform.

We next sought to determine how this putative IRES sequence can be stimulated. IRESs were found to be activated in tumor cells continually subjected to diverse stress conditions of the tumor microenvironment [32, 33]. Furthermore, accumulating evidence argues for the presence of chronic stress of endoplasmic reticulum or unfolded protein response (UPR) in different types of cancers, including breast cancer (for a recent review, see [34]). Given the preferential shift towards cap-independent mRNA translation during UPR [35], we hypothesized that endoplasmic reticulum stress might stimulate the translation of open-reading frames downstream of the major initiation codon. To address this question, we transduced MDA-MB-231 cells with a bi-cistronic lentivector carrying the cDNA sequence of the A/B domain (amino acids 2–173) of ERα cloned between LucR and LucF (Fig. 3b). In these transduced MDA cells (Fig. 3c) as well as in transduced MCF7 cells (Additional file 1: Figure S5A), the LucF/LucR ratios were found to be significantly increased in response to UPR inducers (i.e., DTT and thapsigargin) (Fig. 3c). These inductions correlated with the observed increase in ERα46 protein levels after stress induction in cells stably transfected with the full-length *ESR1* cDNA (Fig. 3d). As a control, we used siRNA directed against LucR which diminished LucF activity (Additional file 1: Figure S5B and C), demonstrating the absence of either an internal promoter in the intervening sequence or stress-induced cryptic alternative splicing that could have shunted the LucR cistron. Taken together, these data suggest that ERα46 can be produced by stress inducers via an IRES-dependent mechanism.

### ERα46 antagonizes the ERα66-mediated proliferative response of MCF-7 cells to E2 in a dose-dependent manner

**Fig. 4:**
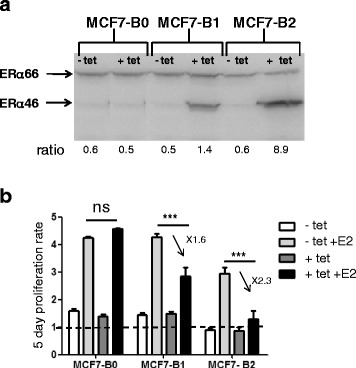
Estrogen receptor alpha (*ERα*)46 antagonizes the 17β-estradiol (*E2*) proliferative response of ERα66 in MCF7 cells. **a** Expression of the ERα46 and ERα66 proteins on MCF7 clones analyzed by Western blotting following tetracycline (*tet*) treatment to induce the ERα46 expression. **b** The proliferative effect of E2 was then analyzed by counting cells after 5 days of stimulation with 10 nM E2. ***P < 0.001. *ns* not significant

Next, we explored the impact of a high expression level of ERα46 on E2-induced proliferation of breast tumor cells using MCF-7 cell lines which were engineered to overexpress a tet-inducible ERα46 (Fig. 4). Proliferation in response to E2 is not influenced by tetracycline treatment as demonstrated using MCF7-B0 sub-clone which expresses an empty vector. By contrast, the proliferation in response to E2 is partially abrogated in the MCF-B1 clone after tetracycline induction (ratio of ERα46/66 close to 1) and almost completely abolished using the MCF7-B2 clone (ratio of ERα46/66 close to 10) which expresses the highest expression level of ERα46. These results indicate that overexpression of the ERα46 isoform inhibited the E2-mediated cell proliferation, with inhibition being proportional to the expression of ERα46.

### Identification of cofactors that differentially interact with ERα66 and ERα46

**Fig. 5:**
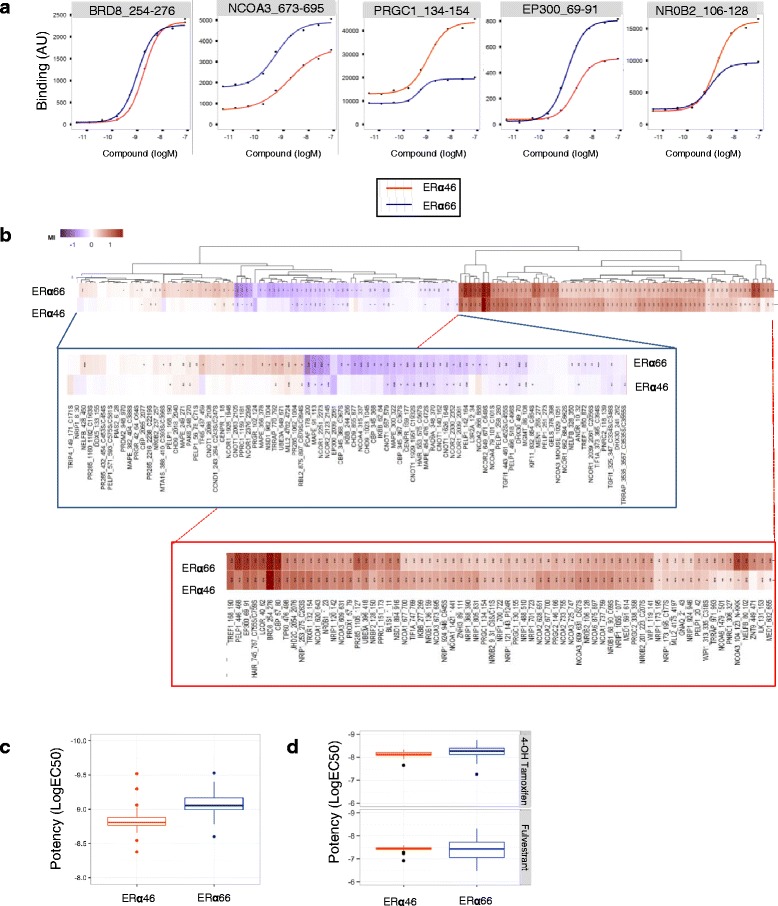
Ligand-specific coregulator binding profiles. Interactions of the estrogen receptor alpha (*ERα*)46 and ERα66 proteins with coregulator motifs were measured using MARCoNI peptide arrays. Interactions were evaluated at different concentrations of E2, ranging from 10^–12^ to 10^–7^ M. **a** Examples of dose-dependent E2-mediated modulation of ERα46 and ERα66 interactions with individual coregulator motifs to illustrate that the two proteins bind to coregulators with differential affinities. **b** Heatmap showing hierarchical clustering (Euclidean distance, average linkage) of E2-mediated interactions (represented as the modulation index (*MI*)) between ERα46 and ERα66 proteins and peptides representing coregulator-derived binding motifs. MI is expressed as log of fold changes relative to vehicle. Zoom outs from the left and the right main clusters are shown below. **c** Boxplot representation of E2 potency for the modulation of coregulator binding of the two isoforms, using EC_50_ values obtained with the curve fit *R* ^2^ > 0.8. **d** Mean of all EC_50_ (logM) values of the ability of fulvestrant and 4-OH-tamoxifen to modulate fully E2-activated coregulator binding to either ERα46 or ERα66

This inhibition of proliferation may occur through the differential recruitment of coregulators by the ERα46 and ERα66 isoforms in the cellular responses induced by E2. To test this hypothesis, we used the MARCoNI assay to characterize the interaction of the two ERα isoforms with 154 unique coregulator-derived motifs, both in their unliganded (apo) conformation or with concentrations of E2 ranging from 10^–12^ to 10^–7^ M, corresponding to full ligand saturation and receptor activation [26]. The resulting overall binding patterns (Additional file 1: Figure S6A and B) indicated that, qualitatively, the receptors bind to the same subset of coregulators, with a clear response of the ERα46 isoform to E2. However, an isoform-selective difference in the binding affinities of both apo and fully-activated receptors was also observed (Fig. 5a). Further analysis of the E2 response curves evidenced that: (i) both isoforms behave similarly for some interactions (with BRD8 for instance); (ii) some peptides bind better to one of the isoforms, for example NCOA3 (also named SRC-3) which has a higher affinity for ERα66 and PRGC1 to ERα46; and (iii) some cofactors bind to both isoforms equally in their apo conformation, but increasing E2 concentrations favor their association with one or the other, as observed for the binding of EP300 to ERα66 or NROB2 to ERα46 (Fig. 5a). The hierarchical clustering of ligand-induced modulation of coregulator interactions was then performed to look for differences and was quantified as the log-fold change in binding (modulation index (MI)) (Fig. 5b). This analysis confirmed that, although qualitatively the overall responses looked generally quite similar, there is a quantitative differential modulation with some selective preference to certain coregulator peptides. Upon E2 binding, an overall increased preference for cofactor binding to ERα66 over ERα46 was observed, as shown in Fig. 5c.

We then investigated the potency of the ERα antagonists 4-OH-tamoxifen and fulvestrant in inhibiting cofactor binding to ERα46 and ERα66 in the presence of E2. The profile of the EC_50_ values for 4-OH-tamoxifen and fulvestrant clearly showed a better efficacy of 4-OH-tamoxifen than fulvestrant in inhibiting E2-induced binding of the receptor isoforms to coregulators (Additional file 1: Figure 6C). However, the potencies of these antagonists to inhibit binding to ERα46 and ERα66 were comparable (Fig. 5d). Altogether, these data clearly demonstrate that the two isoforms show some specificity and heterogeneity in terms of their binding to coregulators.

### Differential gene expression response to E2 mediated by ERα46 and ERα66

**Fig. 6:**
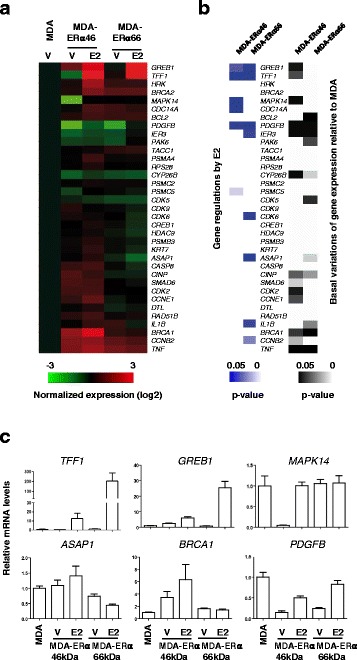
Differential gene expression and response to 17β-estradiol (*E2*) in MDA-ERα46 and MDA-ERα66 cells. **a** The relative expression of a subset of genes was evaluated in MDA, MDA-ERα46, and MDA-ERα66 cells by quantitative RT-PCR following a 4-h treatment with E2 or ethanol as vehicle (*V*). Data were normalized to those obtained for the *Rplp0* gene. Results shown within the heatmap are expressed as the mean log2 of these expression values normalized to those obtained in the control MDA cell line. **b**. Significant gene regulations by E2 in MDA-ERα46 and MDA-ERα66 cells or significant different basal gene expression between naïve MDA cells and estrogen receptor (*ER*) isoforms expressing cells are shown as heatmaps of *P* values. **c**. Examples of gene expression data obtained on six genes

In order to evaluate the impact of differences in coregulator affinity between the two ERα isoforms in terms of transcriptional regulation, we aimed at determining the expression of some target genes in MDA-ERα46 and MDA-ERα66 cells in response to E2. To directly assess the correlation between these events and cell proliferation, we selected a set of genes for their known association with this process, some of them also described as regulated by E2 in MCF7 breast cancer cells or MDA-ERα66 cells (Additional file 1: Figure S7) [36, 37]. The data (Fig. 6 and Additional file 1: Figure S8) indicate that the majority of the tested genes are differentially regulated in MDA-ERα46 and MDA-ERα66 cells. While some genes were found to be regulated by E2 in MDA cells expressing either ERα66 or ERα46, albeit at higher levels for the ERα66 (*GREB1*, *TFF1*, and *PDGFB)*, some genes were specifically regulated by the ERα46 (*MAPPK14* and *CDC14A*) or the ERα66 (*IER3*, *CDK6*, *ASAP1*, *IL1B*, and *CCNB2*). Moreover, the basal levels of transcription were also differentially affected by the expression of these isoforms as compared to naïve MDAwt cells. Indeed, some genes were specifically affected by either the ERα66 (*CDK5* and *IL1B*), or the ERα46 (*GREB1*, *TFF1*, *MAPK14*, *CDK2*, and *CCNE1*) but also by both isoforms (*PDGFB*, *IER3*, *BRCA1*, and *TNF*). Altogether, these data clearly demonstrate that ERα46 and ERα66 have different transcriptional activities.

## Discussion

The work reported here aimed to analyze the expression levels and characteristics of the overlooked ERα46 isoform in breast tumor samples. We have clearly shown that ERα46 is expressed in the majority of human breast tumors tested (more than 70%) with highly variable expression levels, sometimes even more abundant than ERα66. Importantly, the ERα46/ERα66 expression ratio negatively correlated with tumor grade: poorly-differentiated tumors (of higher grade and larger size) presented lower amounts of ERα46. These data indicate that this shorter isoform may have a potential clinical relevance. Unfortunately, since this retrospective study started in 2011, it is too early to further analyze any correlation between the abundance of ERα46 and overall survival or recurrence of disease. This criterion requires a time period of 15–20 years due to delayed tumor relapses of ERα-positive tumors [38].

A previous study reported the expression of a 47-kDa isoform in human breast cancer that is able to bind to radioactive tamoxifen aziridine, which could be the same as the 46-kDa ERα isoform described here [20]. Using electrophoresis with radiolabeled tamoxifen, the authors found that 49% of tumor samples to express the 67- and the 47-kDa protein entities, whereas 36% contained only the longest form. Our proteomic analysis is the first to definitively identify the band detected by Western blot as an ERα isoform deprived of the A/B domain.

Our results also show that several of the antibodies currently used for the diagnosis of breast cancer are unable to detect the ERα46 isoform. Indeed, the 1D5, 6 F11, and EP1 antibodies are directed against the A/B domain, which is absent in ERα46 (Fig. 6). As a consequence, the hormone-dependent characterization of the tumor, presently performed by immunohistochemistry, may only be based on the expression level of ERα66. This finding highlights the importance of the choice of antibodies used for the diagnosis of breast cancer, which able or not to detect the ERα46 isoform.

Furthermore, we found that ERα46 expression level was related to tumor size, suggesting that expression of the 46-kDa isoform in breast tumors could be associated with a limited tumor growth. Such a hypothesis is supported by previous studies demonstrating that ERα46 antagonizes the proliferative effects induced by ERα66 activation both in vitro in MCF7 cells [17] and SaOS osteosarcoma cells [9], as well as in colorectal tumor tissues [12]. Its expression could therefore maintain a low tumor volume, possibly by stimulating apoptosis [12]. We confirmed these data and also found that increasing the amount of ERα46 in MCF7 cells decreases their proliferative response to E2 in a dose-dependent manner. Importantly, other studies have linked the N-terminal region of ERα with cell proliferation. Merot et al. [39] used in vitro systems to show that the respective contribution of AF-1 and AF-2 towards ERα transcriptional activity varies upon the stage of cell differentiation. This key role of AF-1 was also demonstrated physiologically in the uterus, a tissue that is highly sensitive to the proliferative actions of E2 in vivo. Indeed, it was shown that E2 had no proliferative action on uterine epithelial cells in *ERα-AF1* ^*0*^ mice, which express an AF-1-deficient 49-kDa ERα isoform [19]. ERα has been described as being at the crossroads of paracrine or autocrine growth factor and endocrine estrogenic signaling [40], and its activity can be controlled in the absence of E2 through phosphorylation cascades induced by insulin-like growth factor (IGF)-1, epidermal growth factor (EGF), or fibroblast growth factor (FGF)-2. Importantly, most of the residues of ERα that have so far been implicated in these E2-independent responses or in the modulation of ERα activities in response to growth factor signaling are located within the N-terminal region of the protein and constitute an intrinsic part of AF-1 [41]. Altogether, these data support the hypothesis that AF-1 is the region of ERα required for cell proliferation, and that its absence in the ERα46 isoform is likely to confer specific properties to this protein compared to the ERα66 isoform.

In our study, we also analyzed the ability of the two isoforms to bind cofactors using the MARCoNI assay, and found that the two isoforms show some heterogeneity in terms of binding to coregulators. The ability of the two apo-ERα isoforms to recruit transcription factors to the *pS2/TFF1* promoter was previously compared by Re-ChIP experiments [22]. This study identified that ERα46 specifically recruited components of the Sin3 repressive complex (NCOR/SMRT) to the *TFF1* promoter in the absence of E2. This was associated with specific inhibition of the basal transcription of the *TFF1* gene by the ERα46 isoform. More recently, the quaternary structure of a biologically active ERα-coactivator complex on DNA has been determined by cryoelectron microscopy [42]. This study showed the location of the AF-1 domain in the complex, which supports a role in the recruitment of the coactivator SRC-3. Interestingly, in our assay we also found a stronger binding of ERα66 to some peptides derived from SRC-3 (Fig. 5b). In contrast, the ERα46 isoform was found to bind to NRB02 better than the ERα66 isoform. NRB02 acts as a negative regulator of receptor-dependent signaling pathways [43]. These data therefore underline the importance of the AF-1 domain for full transcriptional activation of the ERα-coactivator complex. Indeed, our study also demonstrates a differential gene expression induced by ERα46 or ERα66 at the basal level but also in response to E2. Interestingly, among these differentially regulated genes, ERα46 specifically upregulated the *MAPK14* and *CDC14* genes in the presence of E2 (respectively 20- and 1.7-fold) as opposed to the ERα66 isoform. These genes are implicated in the suppression of the cell proliferation [44, 45] and these regulations may at least partly explain the reduced E2-mediated proliferative response observed when ERα46 is co-expressed. Although not significant, the originally identified proapoptotic *HRK* gene [46] also exhibited a slight tendency to be specifically regulated by the ERα46 (twofold, *P* = 0.09).

Our observations raise the hypothesis that the presence of the short ERα46 isoform in breast tumors could indicate a more favorable prognosis. Such an assessment is also supported by the study of Klinge et al. who indicated that almost 40% of patients developing a secondary tamoxifen resistance exhibit a reduced expression of ERα46 [13]. This supports the idea that endocrine resistance is associated with a decreased expression of ERα46 and thus with poor breast cancer prognosis. Subtle interactions between these isoforms could influence the action of selective estrogen receptor modulators (SERMs) against tumor growth and metastasis. Interestingly, tamoxifen antagonizes the AF-2 of both the ERα66 and ERα46 isoforms, but at the same time acts as an agonist on AF-1 of ERα66 in a tissue-dependent manner. Due to the lack of AF-1, tamoxifen cannot elicit such an action on ERα46. As shown in our analysis with the MARCoNI assay, ERα46 appears to be as potent as the ERα66 in dissociating coregulatory binding in response to tamoxifen or fulvestrant. However, since these interactions are very complex, further investigations are needed in tumor samples in vivo.

**Fig. 7:**
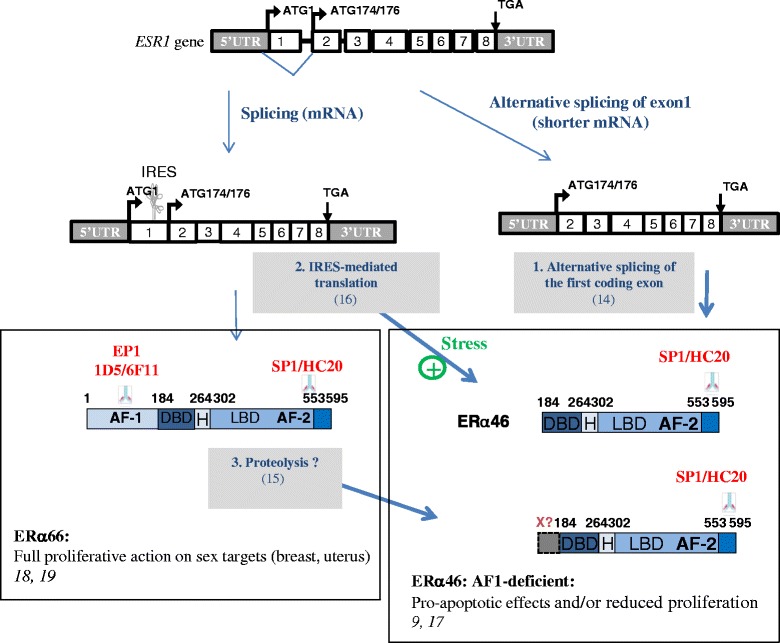
Possible mechanisms to generate the estrogen receptor alpha (*ERα*)46 and epitope mapping of the antibodies used in this work. ERα46 can be generated either by alternative splicing of the first coding exon [16], by internal ribosomal entry site (*IRES*)-dependent translation from the full-length *ESR1* transcript [14], or by proteolysis of ERα66 by as-yet unknown proteases [15]. It should be emphasized that these three different mechanisms could concur to generate the ERα46 isoform, which, to the best of our knowledge, is a unique feature among proteins. *AF* activation function, *DBD* DNA-binding domain, *H* Hinge domain, *LBD* ligand-binding domain

Altogether, these data point out the importance of the expression of both ERα isoforms in breast tumors. In the absence of an ERα46-specific antibody, automated immunoblot analyses would be necessary to render ERα46 detection practically feasible in breast cancer diagnosis. However, it cannot be ruled out that new techniques based on structural properties of the two estrogen receptors could appear in the future [47]. Further characterization of ERα46 will then be needed to refine both prognosis and therapy. Although the exact mechanisms accounting for the expression of the ERα46 isoform still remain to be clarified, three potential processes have been identified: (i) alternative splicing could generate an mRNA deficient in the nucleotide sequence corresponding to exon 1 encoding the A/B domain [14]; (ii) proteolysis, as has also been suggested in human breast tumors [15] and in the mouse uterus [48]; and (iii) an IRES located within the full length mRNA could allow the initiation of translation at a downstream ATG which encodes methionine 174 in the human ERα66 [16] (Fig. 7). Unfortunately, our proteomic approach did not identify peptides close to this initiation codon. One potential explanation for this is the presence of lysine and arginine residues (target residues for trypsin) in the vicinity to the two potential initiation codons (KGS**M**A**M**ESAKETRY). The length of the peptides generated after complete trypsin digestion may be too short to be identified despite the high dynamic range of the nano-LC-MS/MS system used for the proteomic analysis. At this stage, it is not possible to determine the respective roles of the different mechanisms of ERα46 generation. However, we provide evidence that an IRES-dependent alternative translational initiation under stress conditions could lead to the generation of ERα46. This is further supported by the association of the ERα mRNA, along with other IRES-containing mRNAs, to polysomes in apoptotic MCF7 cells in which cap-dependent translation is repressed [49]. Moreover, 4E-BP1, a negative regulator of cap-dependent mRNA translation, was found to be overexpressed in breast tumors compared to healthy epithelium, suggesting that translational mechanisms such as IRES might be active [50]. Interestingly, genes such as such as Apaf-1, DAP5, CHOP, p53, etc., that are also selectively translated by an IRES-driven mechanism, allow the cells to fine-tune their responses to cellular stress and, if conditions for cell survival are not restored, to proceed with final execution of apoptosis [51]. Although the significance of induction of ERα46 by cellular stress remains unknown, this isoform of ERα could be part of an orchestrated IRES-driven response, and contribute to slowing down of the proliferative response to E2.

## Conclusions

This study demonstrates that a shorter ERα46 isoform previously ignored in diagnosis is frequently expressed in ERα-positive breast tumors, as revealed by Western blot analysis. Careful attention should therefore be taken in the choice of antibodies used for immunohistochemistry as several do not to detect the expression of the ERα46 isoform. We have demonstrated that this shorter isoform can differentially bind to coregulators in response to E2 which might modulate the transcriptional hormonal response. This highlights the potential importance of this shorter isoform in E2 signaling and its antiproliferative actions in breast cancer. We indeed found a clear inverse correlation between tumor size and ERα46 levels. Thus, due to the importance of ERα and hormonal treatments in the management of breast cancers, ERα46 expression should now be further studied.

## Additional file

Additional file 1: Figure S1. Clinical parameters of the breast tumor samples. *IDC* invasive ductal carcinoma, *ILC* invasive lobular carcinoma. **Figure S2.** Expression of Flag-ERα36 in transiently transfected MDA-MB231 as detected by immunocytochemistry using an anti-Flag antibody. **Figure S3.** Analysis of potential correlation between the clinical parameters of the breast tumor samples and the expression of ERα46 isoform. A significant *P* value (indicated in red) was only found between ERα46 expression and HER-2 positive breast tumors. *IDC* invasive ductal carcinoma, *ILC* invasive Lobular Carcinoma. **Figure S4.** Results of the proteomic analysis of the ERα46 protein detected in tumor samples. A) Western blot with the SP1 antibody obtained after immunoprecipitation of ERα with HC20 antibody in two human tumors overexpressing the putative ERα46 isoform. B and C) Sequence coverage obtained from the peptides identified by proteomic analysis shown in bold red on ERα66 and on ERα46 isoforms M (methionine): putative translational start sites generating the ERα46 isoform. **Figure S5.** The stress-induced increase in LucF activity is reproducible in MCF7 cells (A) and is not due to the generation of mono-cistronic LucF transcripts via an internal promoter or cryptic splicing as observed in MDA-Lenti-AB exposed to two siRNAs-lucR (B and C). **Figure S6.** Modulation profiles of the interaction of ERα46 (red) and ERα66 (blue) with coregulators in A) Apo proteins and B) in response to E2 binding. C) Profile of EC_50_ values of 4-OH-tamoxifen- (red) and fulvestrant- (blue) induced modulation of ERα46 and ERα66 coregulators interaction when use in antagonists mode with 6.3 nM E2. **Figure S7.** List of primers used in the expression profiling of target genes. **Figure S8.** Fold-changes (FC) in gene expression± SEM in MDA-ERα46 and MDA-ERα66 cells in response to 4-h treatment with E2 (10^–8^ M). *P* values were determined by Mann-Whitney test. Significant *P* values are indicated in red. (PPTX 939 kb)

## Abbreviations

AF-1: Activation function 1
AJCC: American Joint Committee on Cancer
ChIP: Chromatin immunoprecipitation
DMEM: Dulbecco’s modified Eagle’s medium
DTT: 1,4-Dithiothreitol
E2: 17β-estradiol
ECL: Enhanced chemiluminescence
EDTA: Ethylenediaminetetraacetic acid
EGF: Epidermal growth factor
ER: Estrogen receptor
ERE: Estrogen responsive element
FCS: Fetal calf serum
FGF: Fibroblast growth factor
HER2: Human epidermal growth factor receptor-2
HRP: Horseradish peroxidase
IGF: Insulin-like growth factor
IRES: Internal ribosomal entry site
LC-MS/MS: Liquid chromatography coupled to tandem mass spectrometry
LucF: Firefly luciferase
LucR: Renilla luciferase
MARCoNI: Microarray Assay for Real-time Coregulator-Nuclear receptor Interaction
PBS: Phosphate-buffered saline
PCR: Polymerase chain reaction
RT: Reverse transcription
SERM: Selective estrogen receptor modulator
UPR: Unfolded protein response
