# Supplementary material for: The AF-1-deficient estrogen receptor ERα46 isoform is frequently expressed in human breast tumors
Source: Breast Cancer Res. 2016 Dec 7;18:123. doi: 10.1186/s13058-016-0780-7 (PMC5142410; doi:10.1186/s13058-016-0780-7)
Supplement: Additional file 1: Figure S1. — Clinical parameters of the breast tumor samples. IDC invasive ductal carcinoma, ILC invasive lobular carcinoma. Figure S2. Expression of Flag-ERα36 in transiently transfected MDA-MB231 as detected by immunocytochemistry using an anti-Flag antibody. Figure S3. Analysis of potential correlation between the clinical parameters of the breast tumor samples and the expression of ERα46 isoform. A significant P value (indicated in red) was only found between ERα46 expression and HER-2 positive breast tumors. IDC invasive ductal carcinoma, ILC invasive Lobular Carcinoma. Figure S4. Results of the proteomic analysis of the ERα46 protein detected in tumor samples. A) Western blot with the SP1 antibody obtained after immunoprecipitation of ERα with HC20 antibody in two human tumors overexpressing the putative ERα46 isoform. B and C) Sequence coverage obtained from the peptides identified by proteomic analysis shown in bold red on ERα66 and on ERα46 isoforms M (methionine): putative translational start sites generating the ERα46 isoform. Figure S5. The stress-induced increase in LucF activity is reproducible in MCF7 cells (A) and is not due to the generation of mono-cistronic LucF transcripts via an internal promoter or cryptic splicing as observed in MDA-Lenti-AB exposed to two siRNAs-lucR (B and C). Figure S6. Modulation profiles of the interaction of ERα46 (red) and ERα66 (blue) with coregulators in A) Apo proteins and B) in response to E2 binding. C) Profile of EC50 values of 4-OH-tamoxifen- (red) and fulvestrant- (blue) induced modulation of ERα46 and ERα66 coregulators interaction when use in antagonists mode with 6.3 nM E2. Figure S7. List of primers used in the expression profiling of target genes. Figure S8. Fold-changes (FC) in gene expression± SEM in MDA-ERα46 and MDA-ERα66 cells in response to 4-h treatment with E2 (10–8 M). P values were determined by Mann-Whitney test. Significant P values are indicated in red. (PPTX 939 kb) [file 13058_2016_780_MOESM1_ESM.pptx]

## Slide 1
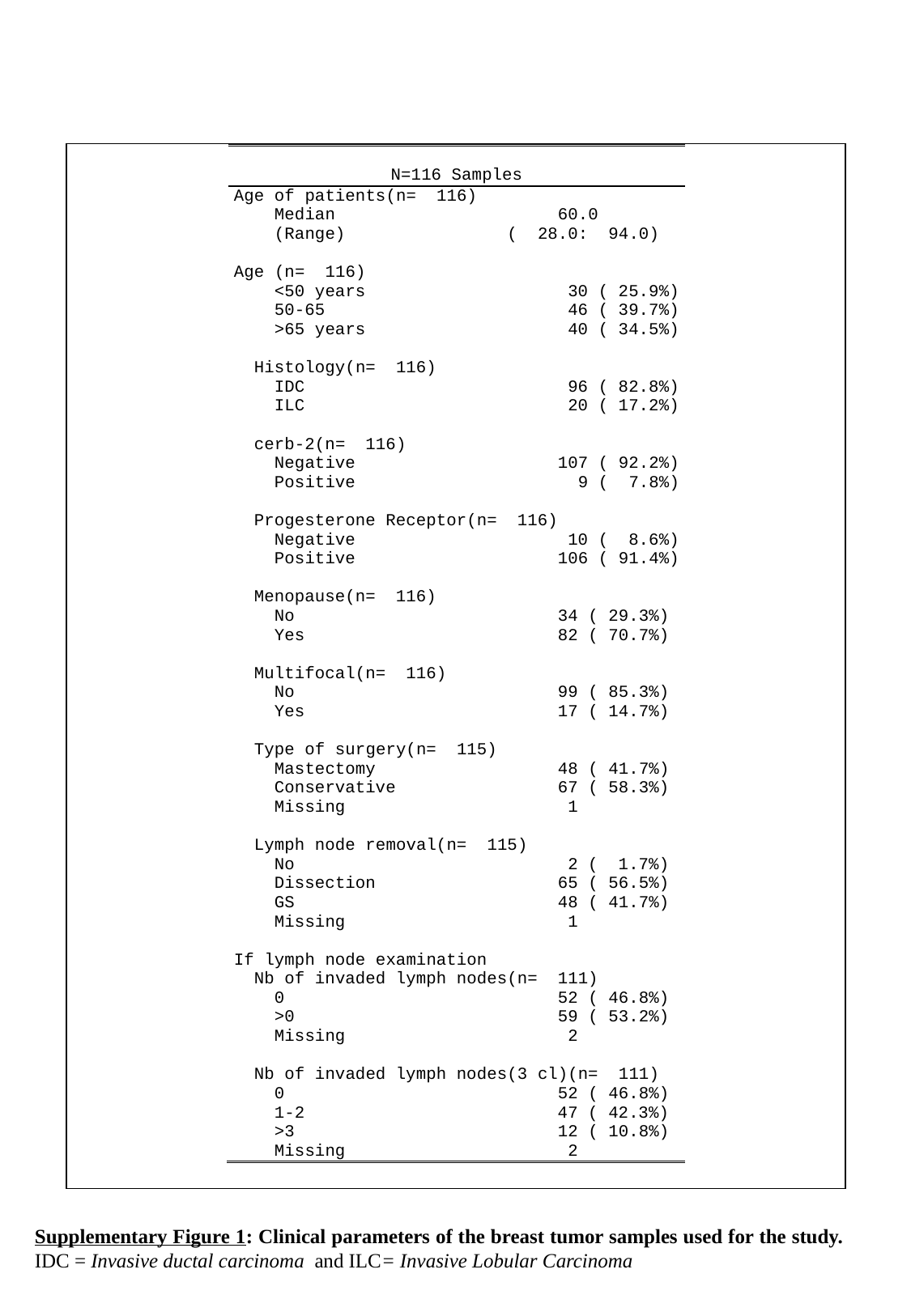

Supplementary Figure 1: Clinical parameters of the breast tumor samples used for the study. IDC = Invasive ductal carcinoma and ILC= Invasive Lobular Carcinoma

## Slide 2
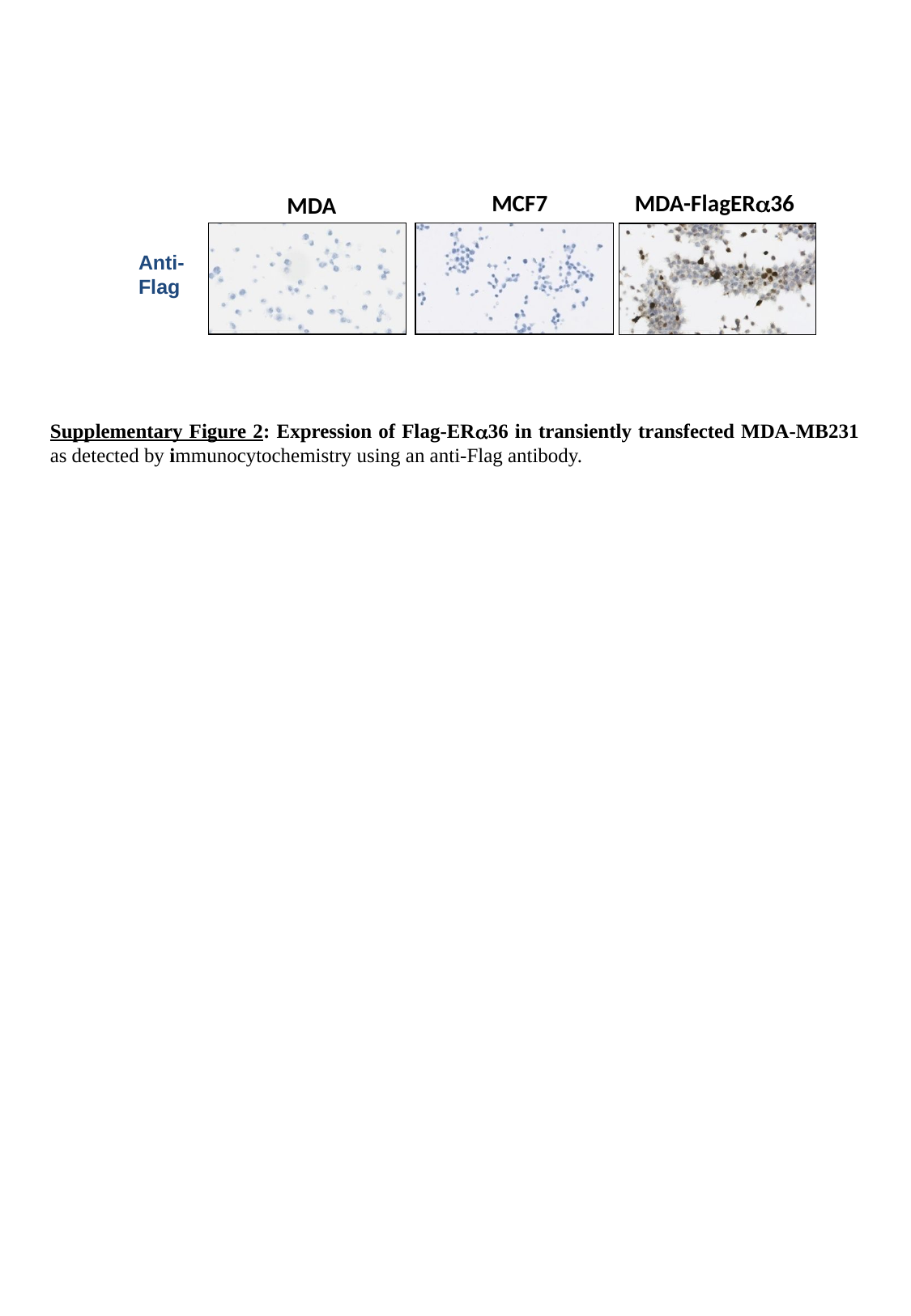

MCF7
MDA-FlagERα36
MDA
Anti-
Flag
Supplementary Figure 2: Expression of Flag-ERα36 in transiently transfected MDA-MB231 as detected by immunocytochemistry using an anti-Flag antibody.

## Slide 3
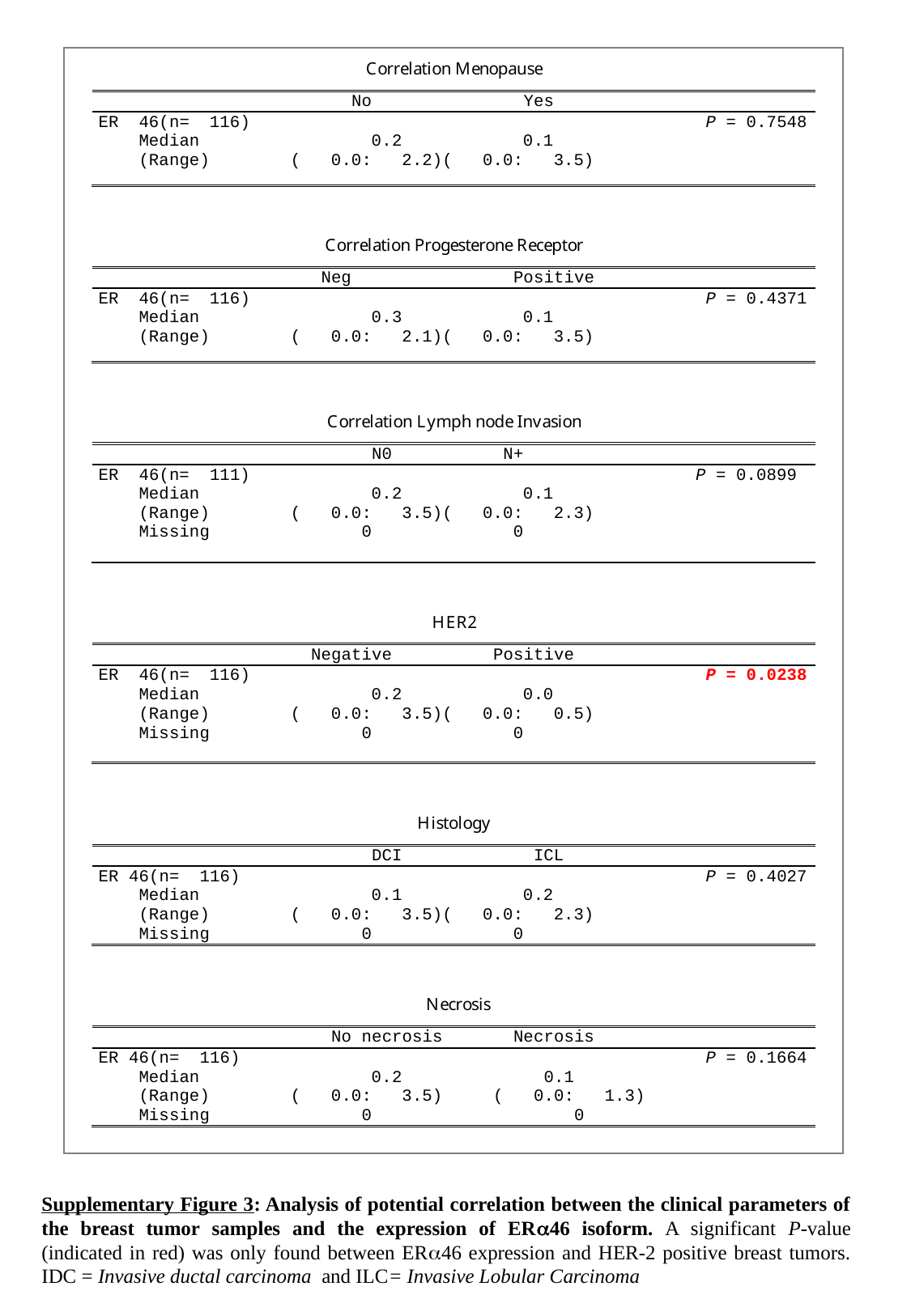

Supplementary Figure 3: Analysis of potential correlation between the clinical parameters of the breast tumor samples and the expression of ERα46 isoform. A significant P-value (indicated in red) was only found between ERα46 expression and HER-2 positive breast tumors. IDC = Invasive ductal carcinoma and ILC= Invasive Lobular Carcinoma

## Slide 4
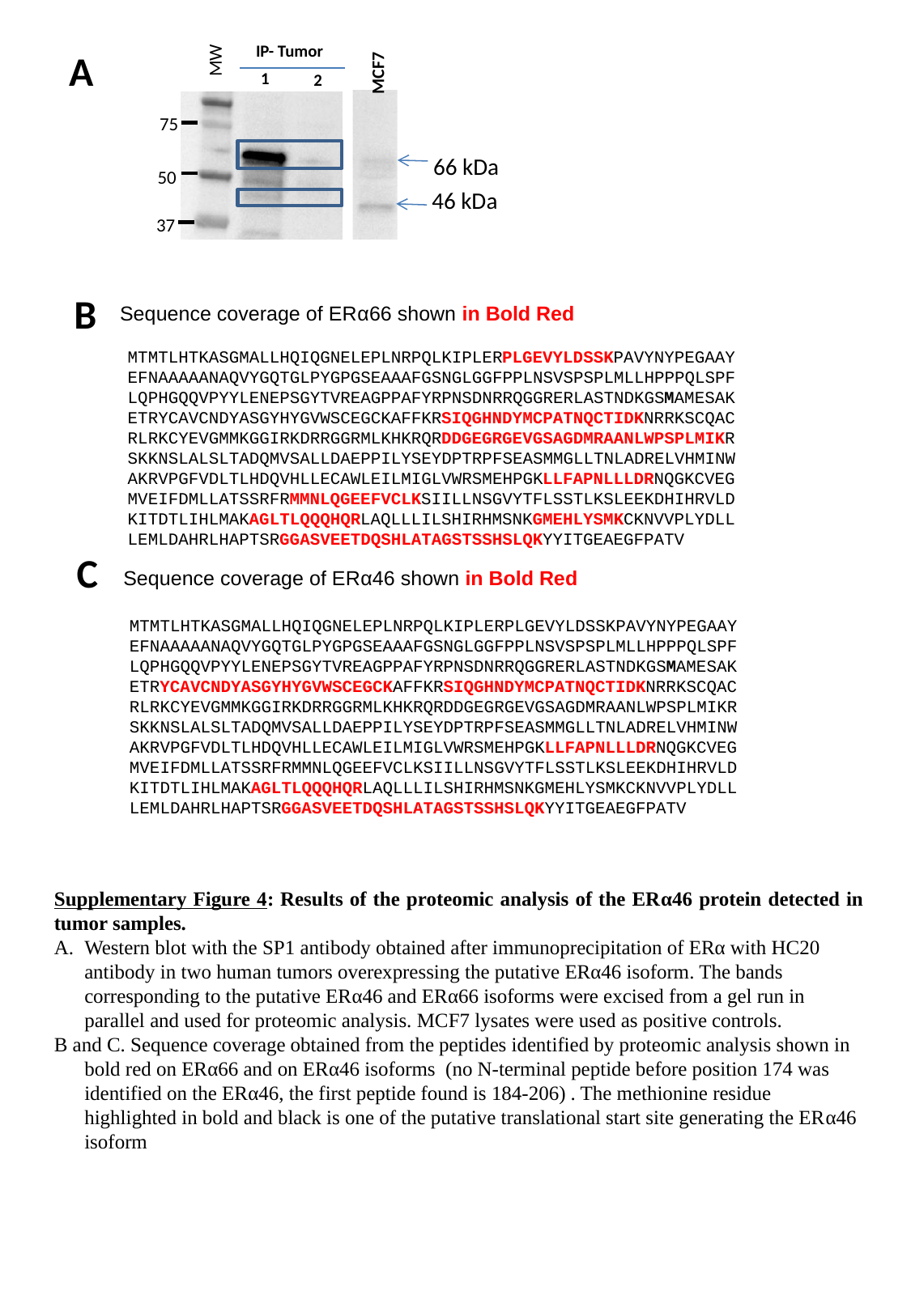

IP- Tumor
MCF7
2
75
66 kDa
50
46 kDa
37
A
MW
1
B
Sequence coverage of ERα66 shown in Bold Red
MTMTLHTKASGMALLHQIQGNELEPLNRPQLKIPLERPLGEVYLDSSKPAVYNYPEGAAY
EFNAAAAANAQVYGQTGLPYGPGSEAAAFGSNGLGGFPPLNSVSPSPLMLLHPPPQLSPF
LQPHGQQVPYYLENEPSGYTVREAGPPAFYRPNSDNRRQGGRERLASTNDKGSMAMESAK
ETRYCAVCNDYASGYHYGVWSCEGCKAFFKRSIQGHNDYMCPATNQCTIDKNRRKSCQAC
RLRKCYEVGMMKGGIRKDRRGGRMLKHKRQRDDGEGRGEVGSAGDMRAANLWPSPLMIKR
SKKNSLALSLTADQMVSALLDAEPPILYSEYDPTRPFSEASMMGLLTNLADRELVHMINW
AKRVPGFVDLTLHDQVHLLECAWLEILMIGLVWRSMEHPGKLLFAPNLLLDRNQGKCVEG
MVEIFDMLLATSSRFRMMNLQGEEFVCLKSIILLNSGVYTFLSSTLKSLEEKDHIHRVLD
KITDTLIHLMAKAGLTLQQQHQRLAQLLLILSHIRHMSNKGMEHLYSMKCKNVVPLYDLL
LEMLDAHRLHAPTSRGGASVEETDQSHLATAGSTSSHSLQKYYITGEAEGFPATV
C
Sequence coverage of ERα46 shown in Bold Red
MTMTLHTKASGMALLHQIQGNELEPLNRPQLKIPLERPLGEVYLDSSKPAVYNYPEGAAY
EFNAAAAANAQVYGQTGLPYGPGSEAAAFGSNGLGGFPPLNSVSPSPLMLLHPPPQLSPF
LQPHGQQVPYYLENEPSGYTVREAGPPAFYRPNSDNRRQGGRERLASTNDKGSMAMESAK
ETRYCAVCNDYASGYHYGVWSCEGCKAFFKRSIQGHNDYMCPATNQCTIDKNRRKSCQAC
RLRKCYEVGMMKGGIRKDRRGGRMLKHKRQRDDGEGRGEVGSAGDMRAANLWPSPLMIKR
SKKNSLALSLTADQMVSALLDAEPPILYSEYDPTRPFSEASMMGLLTNLADRELVHMINW
AKRVPGFVDLTLHDQVHLLECAWLEILMIGLVWRSMEHPGKLLFAPNLLLDRNQGKCVEG
MVEIFDMLLATSSRFRMMNLQGEEFVCLKSIILLNSGVYTFLSSTLKSLEEKDHIHRVLD
KITDTLIHLMAKAGLTLQQQHQRLAQLLLILSHIRHMSNKGMEHLYSMKCKNVVPLYDLL
LEMLDAHRLHAPTSRGGASVEETDQSHLATAGSTSSHSLQKYYITGEAEGFPATV
Supplementary Figure 4: Results of the proteomic analysis of the ERα46 protein detected in tumor samples.
Western blot with the SP1 antibody obtained after immunoprecipitation of ERα with HC20 antibody in two human tumors overexpressing the putative ERα46 isoform. The bands corresponding to the putative ERα46 and ERα66 isoforms were excised from a gel run in parallel and used for proteomic analysis. MCF7 lysates were used as positive controls.
B and C. Sequence coverage obtained from the peptides identified by proteomic analysis shown in bold red on ERα66 and on ERα46 isoforms (no N-terminal peptide before position 174 was identified on the ERα46, the first peptide found is 184-206) . The methionine residue highlighted in bold and black is one of the putative translational start site generating the ERα46 isoform

## Slide 5
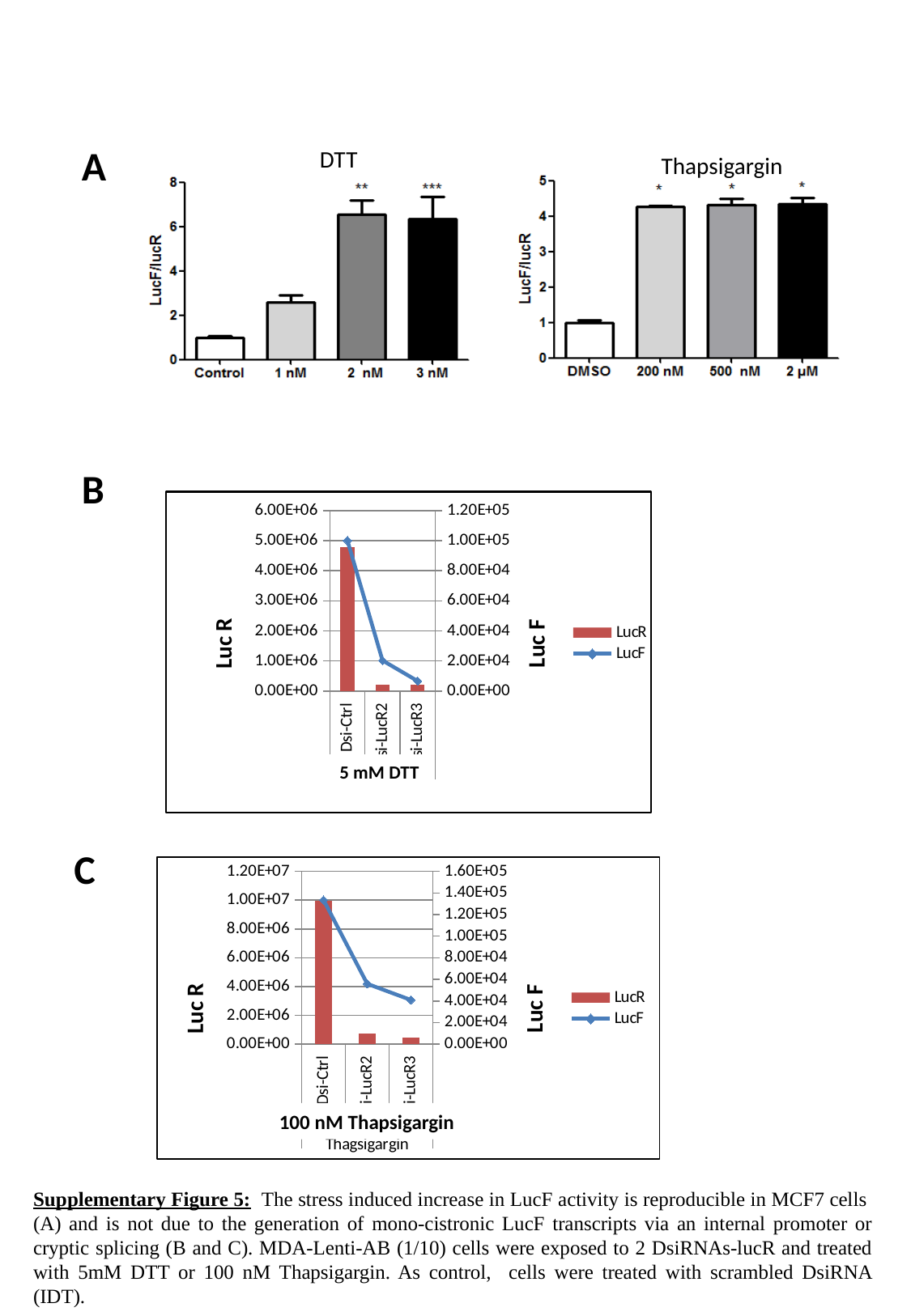

A
DTT
Thapsigargin
B
### Chart
| Category | LucR | LucF |
|---|---|---|
| Dsi-Ctrl | 4777269.0 | 100077.5 |
| Dsi-LucR2 | 197969.5 | 20288.0 |
| Dsi-LucR3 | 200577.0 | 6662.0 |5 mM DTT
C
### Chart
| Category | LucR | LucF |
|---|---|---|
| Dsi-Ctrl | 9966641.75 | 133442.0 |
| Dsi-LucR2 | 724011.5 | 55961.5 |
| Dsi-LucR3 | 465931.0 | 40964.5 |100 nM Thapsigargin
Supplementary Figure 5: The stress induced increase in LucF activity is reproducible in MCF7 cells (A) and is not due to the generation of mono-cistronic LucF transcripts via an internal promoter or cryptic splicing (B and C). MDA-Lenti-AB (1/10) cells were exposed to 2 DsiRNAs-lucR and treated with 5mM DTT or 100 nM Thapsigargin. As control, cells were treated with scrambled DsiRNA (IDT).

## Slide 6
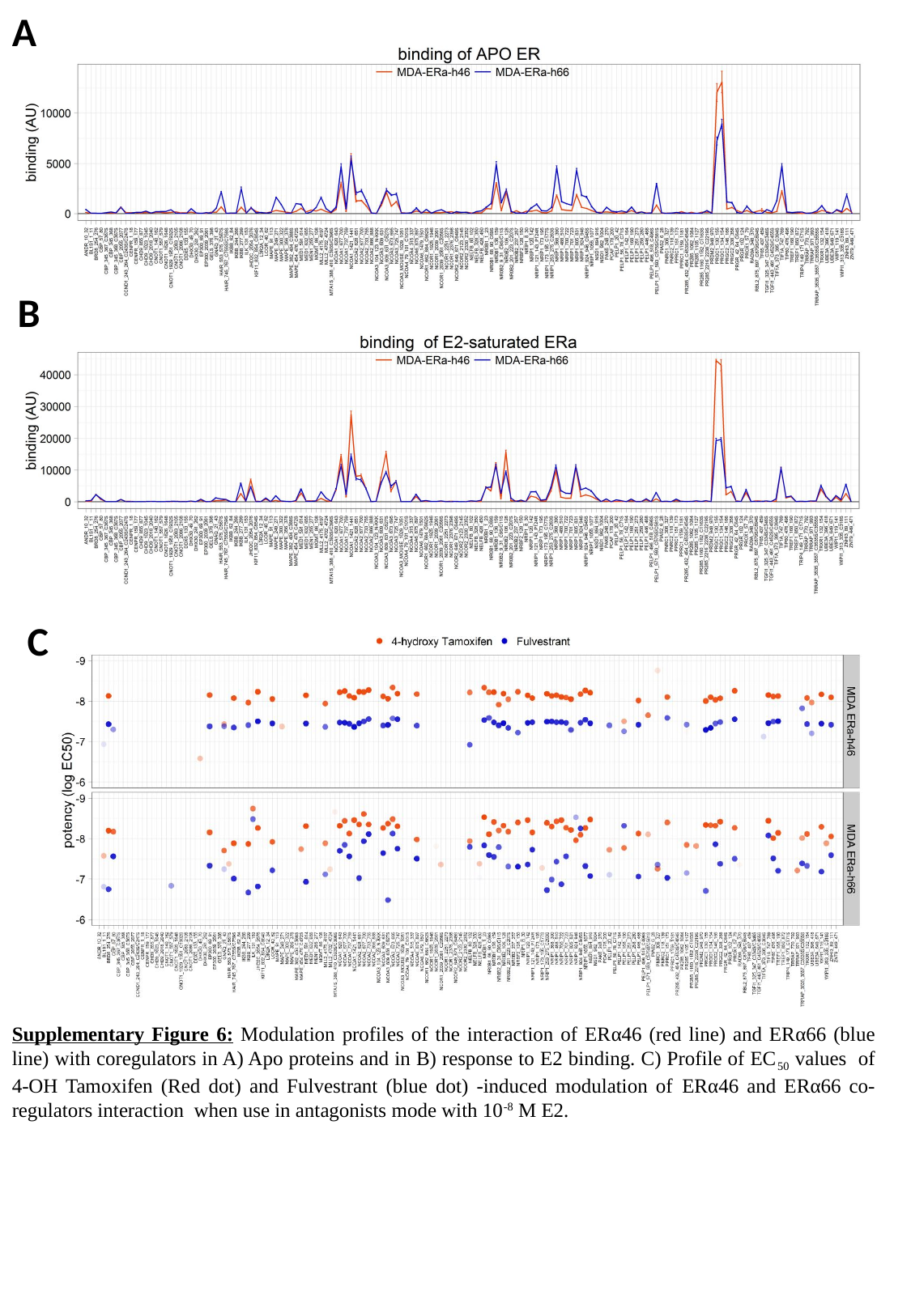

A
B
C
Supplementary Figure 6: Modulation profiles of the interaction of ERα46 (red line) and ERα66 (blue line) with coregulators in A) Apo proteins and in B) response to E2 binding. C) Profile of EC50 values of 4-OH Tamoxifen (Red dot) and Fulvestrant (blue dot) -induced modulation of ERα46 and ERα66 co-regulators interaction when use in antagonists mode with 10-8 M E2.

## Slide 7
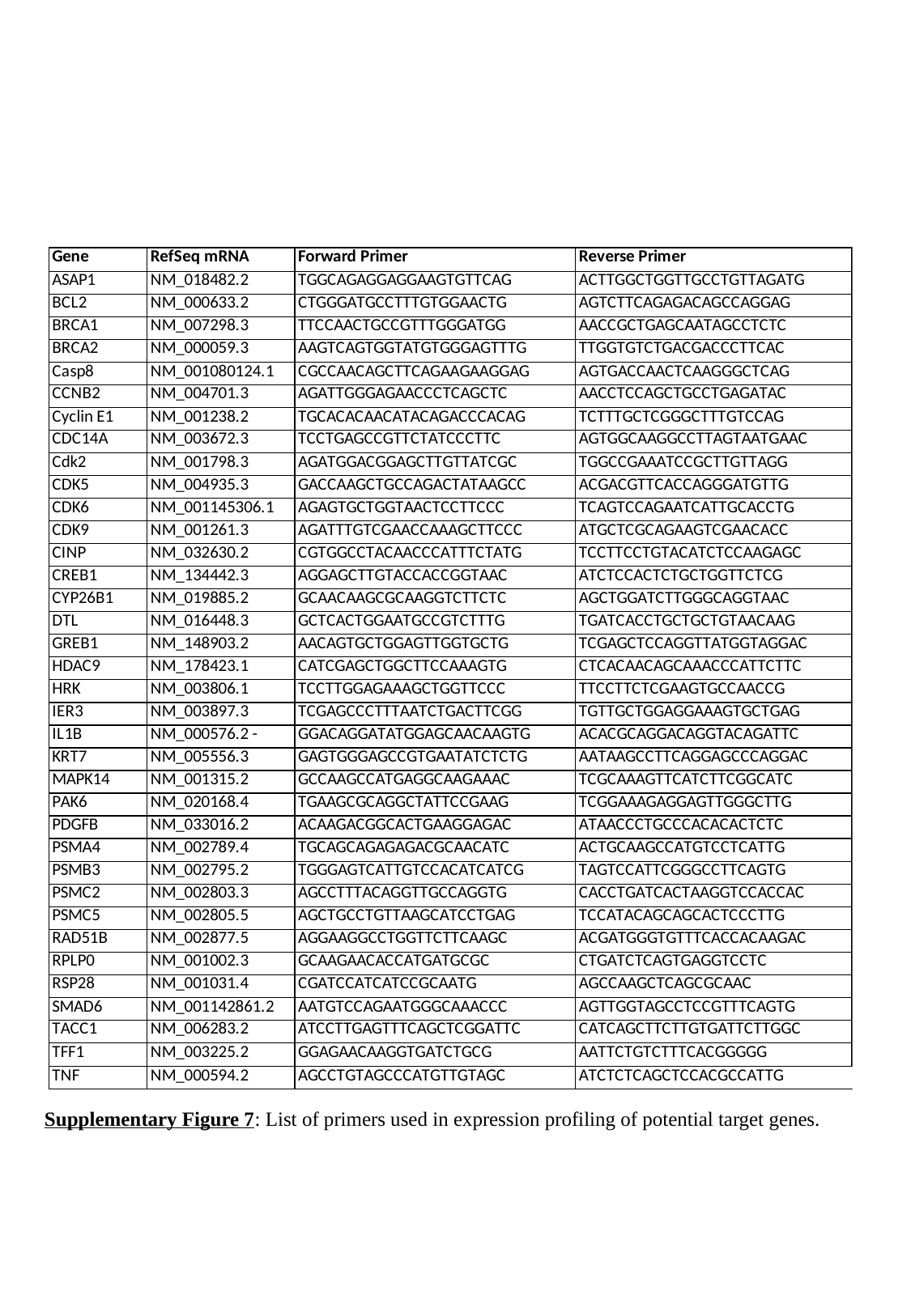

Supplementary Figure 7: List of primers used in expression profiling of potential target genes.

## Slide 8
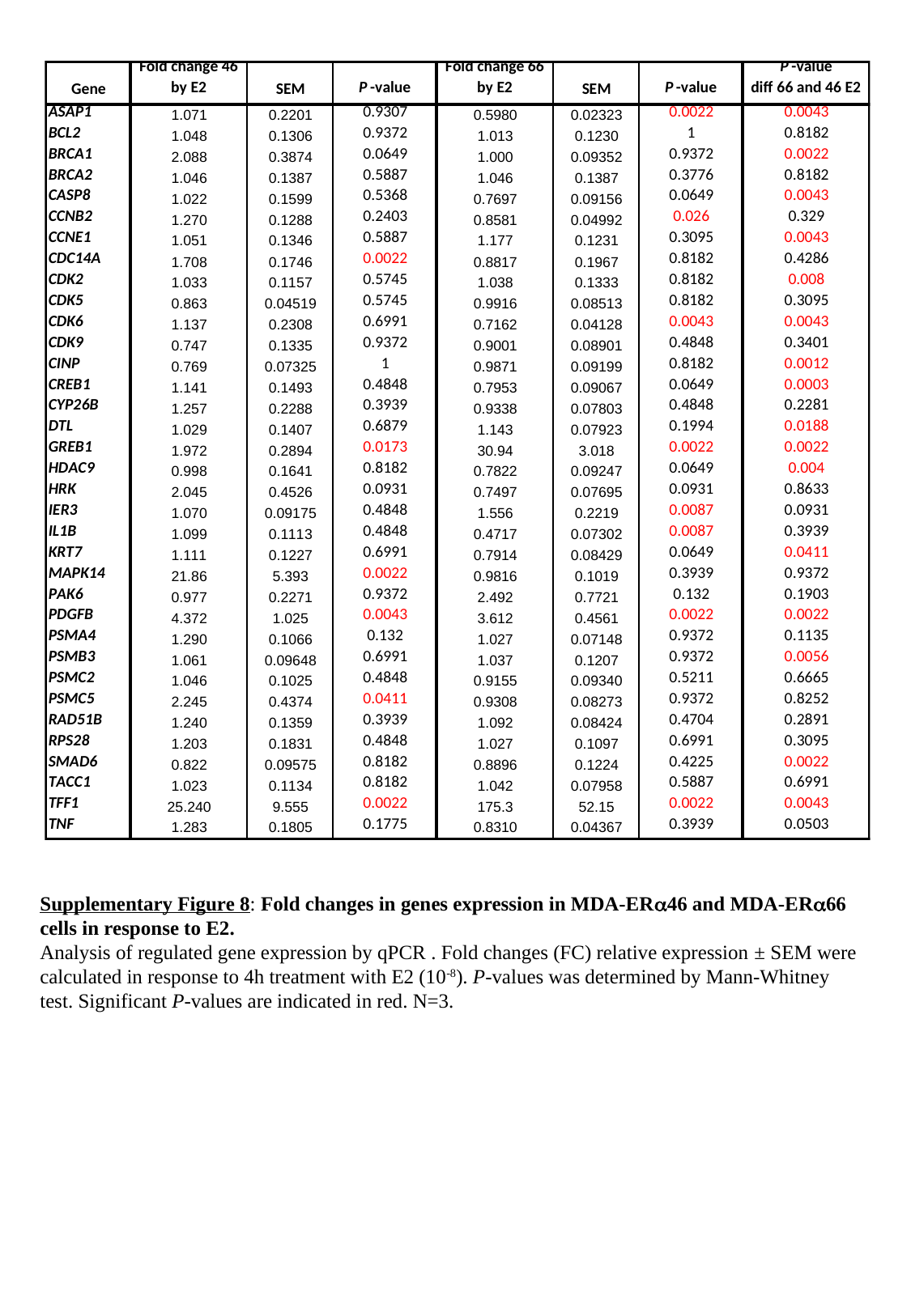

Supplementary Figure 8: Fold changes in genes expression in MDA-ERα46 and MDA-ERα66 cells in response to E2.
Analysis of regulated gene expression by qPCR . Fold changes (FC) relative expression ± SEM were calculated in response to 4h treatment with E2 (10-8). P-values was determined by Mann-Whitney test. Significant P-values are indicated in red. N=3.
